# Survival-associated heterogeneity of marker-defined perivascular cells in colorectal cancer

**DOI:** 10.18632/oncotarget.9632

**Published:** 2016-05-26

**Authors:** Artur Mezheyeuski, Maja Bradic Lindh, Tormod Kyrre Guren, Anca Dragomir, Per Pfeiffer, Elin H. Kure, Tone Ikdahl, Eva Skovlund, Sara Corvigno, Carina Strell, Kristian Pietras, Fredrik Ponten, Jan Mulder, Camilla Qvortrup, Anna Portyanko, Kjell Magne Tveit, Bengt Glimelius, Halfdan Sorbye, Arne Östman

**Affiliations:** ^1^ Department of Oncology-Pathology, Karolinska Institutet, Stockholm, Sweden; ^2^ Department of Pathology, Belarusian State Medical University, Minsk, Belarus; ^3^ Department of Oncology, Oslo University Hospital, Oslo, Norway; ^4^ K.G.Jebsen Colorectal Cancer Research Center, Oslo University Hospital, Oslo, Norway; ^5^ Department of Immunology, Genetics and Pathology, Uppsala University, Uppsala, Sweden; ^6^ Department of Oncology, University of Southern Denmark, Odense, Denmark; ^7^ Department of Cancer Genetics, Institute for Cancer Research, Oslo University Hospital, Oslo, Norway; ^8^ Akershus University Hospital, Lørenskog, Norway; ^9^ School of Pharmacy, University of Oslo and the Norwegian Institute of Public Health, Oslo, Norway; ^10^ Division of Translational Cancer Research, Lund University, Lund, Sweden; ^11^ Department of Neuroscience, Science for Life Laboratory, Karolinska Institutet, Stockholm, Sweden; ^12^ Department of Oncology, Haukeland University Hospital, Bergen, Norway

**Keywords:** PDGFR, perivascular cells, colorectal cancer, tumor stroma, cancer associated fibroblasts

## Abstract

Perivascular cells (PC) were recently implied as regulators of metastasis and immune cell activity. Perivascular heterogeneity in clinical samples, and associations with other tumor features and outcome, remain largely unknown.

Here we report a novel method for digital quantitative analyses of vessel characteristics and PC, which was applied to two collections of human metastatic colorectal cancer (mCRC).

Initial analyses identified marker-defined subsets of PC, including cells expressing PDGFR-β or α-SMA or both markers. PC subsets were largely independently expressed in a manner unrelated to vessel density and size. Association studies implied specific oncogenic mutations in malignant cells as determinants of PC status. Semi-quantitative and digital-image-analyses-based scoring of the NORDIC-VII cohort identified significant associations between low expression of perivascular PDGFR-α and -β and shorter overall survival. Analyses of the SPCRC cohort confirmed these findings. Perivascular PDGFR-α and -β remained independent factors for survival in multivariate analyses.

Overall, our study identified host vasculature and oncogenic status as determinants of tumor perivascular features. Perivascular PDGFR-α and -β were identified as novel independent markers predicting survival in mCRC. The novel methodology should be suitable for similar analyses in other tumor collections.

## INTRODUCTION

Pericytes surround the endothelial cells of small vessels and are embedded in their basement membrane. Through interactions with endothelial cells, pericytes are involved in vessel function and maturation, which includes reciprocal paracrine interaction between endothelial cells and pericytes. Pericyte function is controlled by growth factors including PDGFs, members of the TGFbeta family, sphingosine-1-phosphate-1 (S1P1) and Ang1 (reviewed in [1]). General interest in pericytes has increased based on recent studies showing that subsets of pericytes act as cell-of-origin for fibroblast-like cells in scarring tissue, fibrosis and gliosis [2–6]. Most tissue analyses of pericytes rely on use of one or multiple marker proteins including Platelet-derived growth factor (PDGF) α-tyrosine kinase receptors (PDGFR-α), PDGFR-β, α-SMA, desmin, NG2 and RGS5 [7–10]. Although not systematically investigated, most data suggest that these markers are expressed in a non-overlapping manner and might be linked to yet-to-be-defined functionally relevant pericyte subsets.

A series of recent studies has also highlighted potential important roles of perivascular cells in different aspects of tumor biology. Earlier studies, focusing on tumor growth as end-point, have demonstrated both tumor-stimulatory and -inhibitory effects of increased pericyte coverage, indicating different context-dependent and tumor type-specific mechanisms [11–13]. These findings have recently been expanded by experimental studies, which have linked pericytes to metastasis, immune cell infiltration and efficacy of anti-angiogenic treatment [10, 14–16].

Together these experimental studies provide a strong rational for a more detailed analyses of perivascular status in clinical samples. Analyses of perivascular cells in clinical samples remain scarce, although some early studies have reported associations with survival [7, 10, 17]. Notably, these studies have been limited to the use of a single marker, thus failing to explore the presence of marker-defined subsets. Furthermore, outcome-related studies have relied on semi-quantitative manual scoring methods with limited stringency and accuracy.

This study presents a detailed characterization of perivascular heterogeneity in human colorectal cancer including digital-image-analyses-based quantification. A series of findings are presented including novel candidate determinants of tumor perivascular status and previously un-recognized associations between marker-defined perivascular subsets and survival.

## RESULTS

### Perivascular markers show independent and heterogeneous expression in CRC when analyzed at cellular, vessel or case basis

Results of this study are based on analyses of two independent tumor cohorts which have been analyzed with four perivascular markers and evaluated with two scoring approaches. A summary of the data-set and scoring is presented in Supp. Item 1.

Initial analyses of perivascular cells in CRC aimed at exploring heterogeneity in marker expression at the cellular, vessel and case levels. These analyses focused on the markers PDGFR-β, α-SMA and desmin.

**Figure 1:**
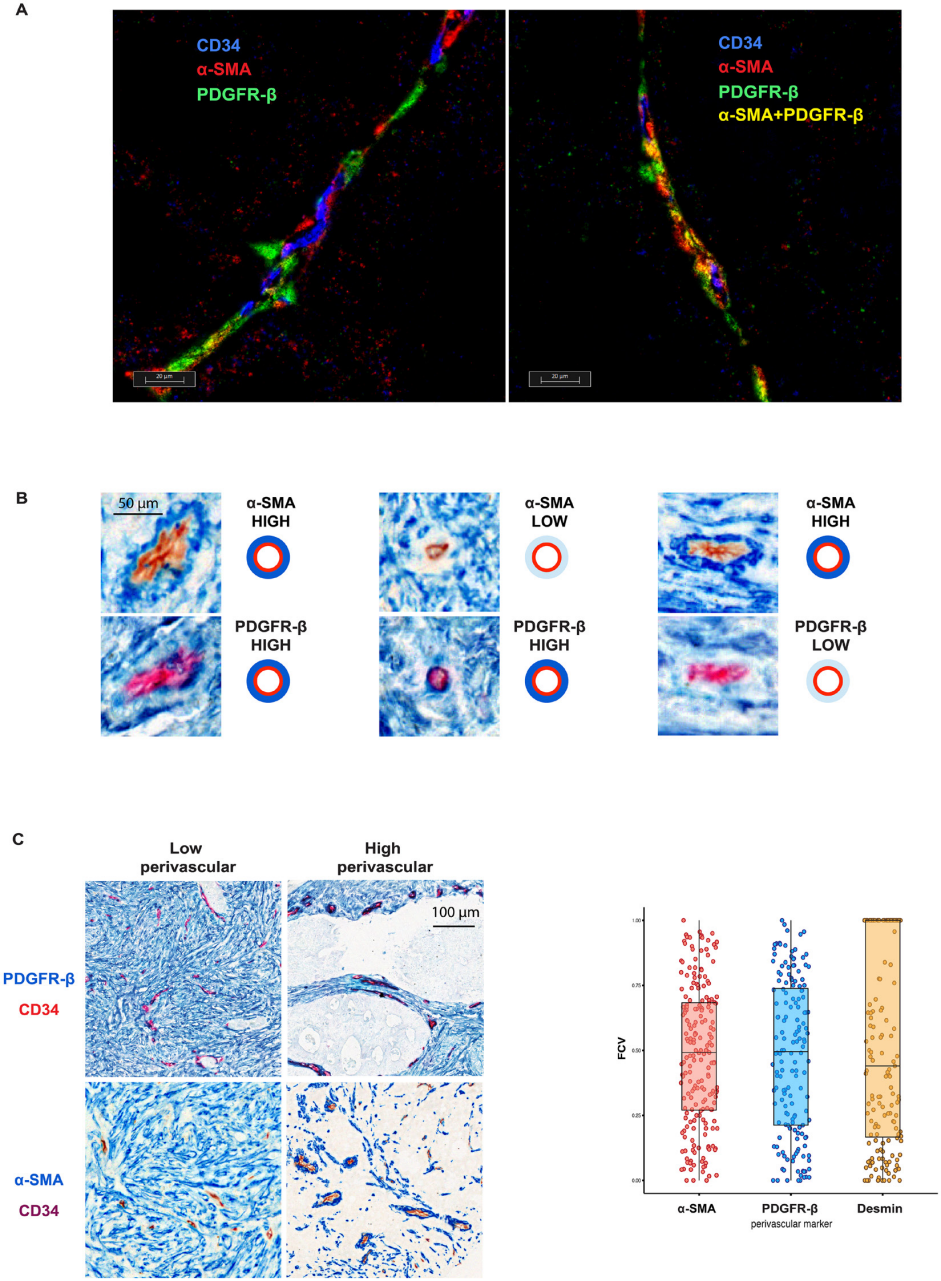
A. Individual perivascular cells express different markers The triple staining with both perivascular cell markers (PDGFR-β (red) or α-SMA (green)) and CD34 (blue) demonstrate presence of perivascular cells of three distinct perivascular cell types: PDGFR-β- and α-SMA-single positive cells and double-positive cells. **B. Individual vessels are characterized by relatively independent perivascular expression of PDGFR-β and α-SMA.** Serial sections of three vessels stained with α-SMA (blue staining, upper panels) or PDGFR-β (blue staining, lower panel) and CD34 (brown or red staining) showing independent perivascular expression of PDGFR-β and α-SMA. Note in left part an α-SMA-high / PDGFR-β-high vessel, in the middle part an α-SMA-low / PDGFR-β –high vessel and in right an α-SMA-high / PDGFR-β-low vessel. **C. Inter-tumoral variation of perivascular marker expression.** Left panel: Representative images of PDGFR-β and α-SMA (blue) double staining with CD34 (red or brown) showing areas of low or high perivascular marker expression. Note different intensity of perivascular staining (blue) in different cases. Right panel: Distribution of the fraction of covered vessels with PDGFR-β, α-SMA and Desmin in Nordic-VII cohort (right panel). The cases of the cohort are characterized by heterogeneous perivascular cell coverage defined by three markers.

For high-resolution analyses of marker-expression, triple immuno-fluorescence staining was done with the endothelial cell marker CD34 and the two of perivascular markers: PDGFR-β and α-SMA. As shown in Figure 1A all three possible marker-defined perivascular cell subsets, i.e. PDGFR-β- and α-SMA-single positive cells and double-positive cells were identified by these analyses.

The next analyses focused on inter-vessel variation in individual tumors. These analyses identified a large degree of inter-vessel variation inside tumors with vessels of the same tumors showing e.g. high or low perivascular PDGFR-β expression which occurred in the absence or presence of α-SMA expression (Figure 1B).

Furthermore, perivascular status was compared in an inter-case manner. As shown at the microphotographs on Figure 1C (left panel) using PDGFR-β and α-SMA as perivascular markers, large tumor areas may be characterized by either low or high marker expression. The large degree of inter-case variations of perivascular α-SMA, PDGFR-β and desmin is illustrated on Figure 1C, right panel. This inter-case variability was also confirmed by semi-quantitative scoring of perivascular PDGFR-β and PDGFR-α (see Supp Item. 2)

### Perivascular status in primary tumors is independent of vessel size and density but associated with BRAF mutation status and perivascular status in normal tissue

The observation of large inter-case variations in perivascular status prompted a set of analyses exploring the potential mechanism(s) causing this variability.

An analytical pipeline was developed for more detailed quantification of marker-defined perivascular cells and vessel characteristics. This digital analyses define vessel diameter, vessel density and produce two perivascular “metrics” for each perivascular marker: perivascular intensity (PVI) and fraction covered vessels (FCV) (for details see Materials and Methods, Supp. Methods and Supp. Items 3). For the validation of the digital quantification two independent observers performed semi-quantitative four-grade scoring of vessel density, vessel diameter and perivascular PDGFR-β status. As shown in Supp. Item 4, digital measurements show high concordance with visual scores.

**Figure 2:**
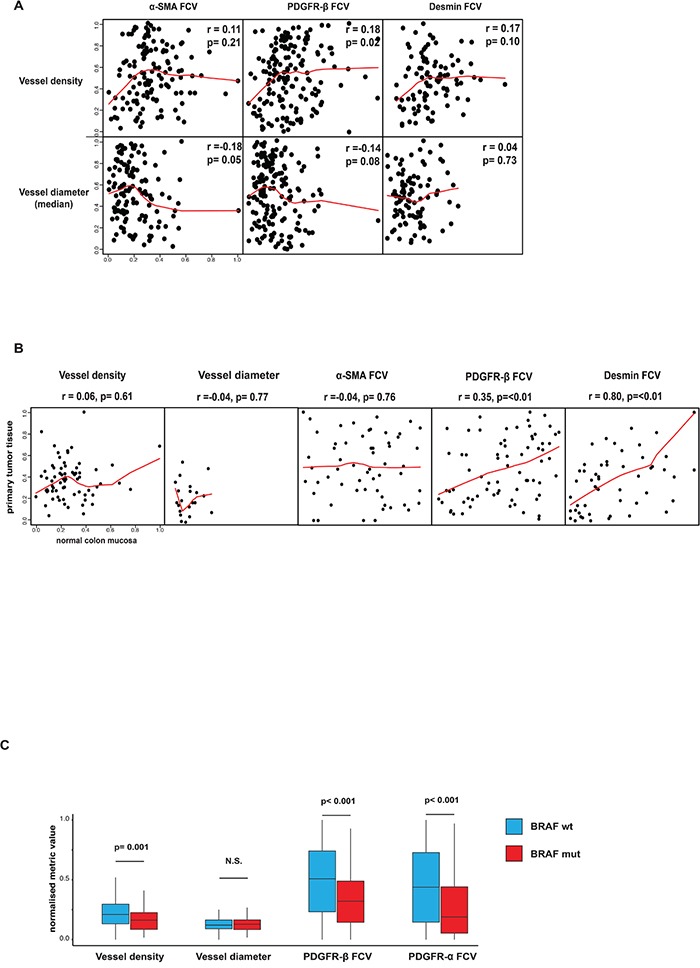
A. Associations between perivascular status and vessel size or vessel density Perivascular α-SMA, PDGFR-β and desmin status was largely independent of both vessel density and vessel size in analyses correlating these features on a case basis. Spearman rank test used for statistical analysis. **B. Associations of vessel size, vessel density or perivascular status between primary tumor and normal colon mucosa.** Perivascular PDGFR-β and desmin status in normal and tumor tissue showed significant correlations. Notably, vessel density, vessel size and perivascular α-SMA did not show this correlation. Horizontal axes show tumor tissue data, vertical axes show normal tissue data. Spearman rank test used for statistical analysis. **C. Associations between perivascular and vascular status BRAF-mutation.** Statistically significant differences were detected regarding perivascular PDGFR-α, PDGFR-β and vessel density in tumors with or without BRAF mutation. Average values of vessel number analyzed with regard to presence of correlation inside one tumor/peritumoral area. p values determined by Mann–Whitney U test.

Relationships between perivascular status and vessel size or vessel density were analyzed in the Nordic VII cohort and the SPCRC cohort. In general, perivascular α-SMA, PDGFR-β and desmin status was largely independent of both vessel density and vessel size in analyses correlating these features on a case basis (Figure 2A). Similar results were obtained when correlation analyses were performed on individual vessels (Supp. Item. 5).

In the case of the Nordic VII cohort, tissue from normal colon was available in a number of cases. Interestingly, perivascular PDGFR-β and desmin but not α-SMA status in normal and tumor tissue showed a significant correlation (Figure 2B). Notably, vessel density and vessel size did not show this correlation (Figure 2B).

BRAF- and KRAS-mutation data from the SPCRC cohort allowed exploratory analyses on potential links between certain oncogenic features and vascular/perivascular status. Interestingly, significant differences with perivascular PDGFR-α, PDGFR-β and vessel density were detected between tumors with or without BRAF mutation (Figure 2C). No associations were detected when KRAS status and perivascular markers or vessel metrics were correlated (data not shown).

Together these analyses identify previously un-recognized associations between perivascular features and host vessel status, and between perivascular features and tumor mutation status.

### Perivascular status predicts survival in the nordic VII cohorts of mCRC as determined by two independent methods

The impact of perivascular marker expression on survival in mCRC has not yet been explored. To investigate this issue, semi-quantitative immunohistochemistry data for perivascular PDGFR-α and PDGFR-β (see Material and Methods and Supp. Item 1 for details) were collected and combined with clinicopathological and survival data from the NORDIC-VII study [18].

**Figure 3:**
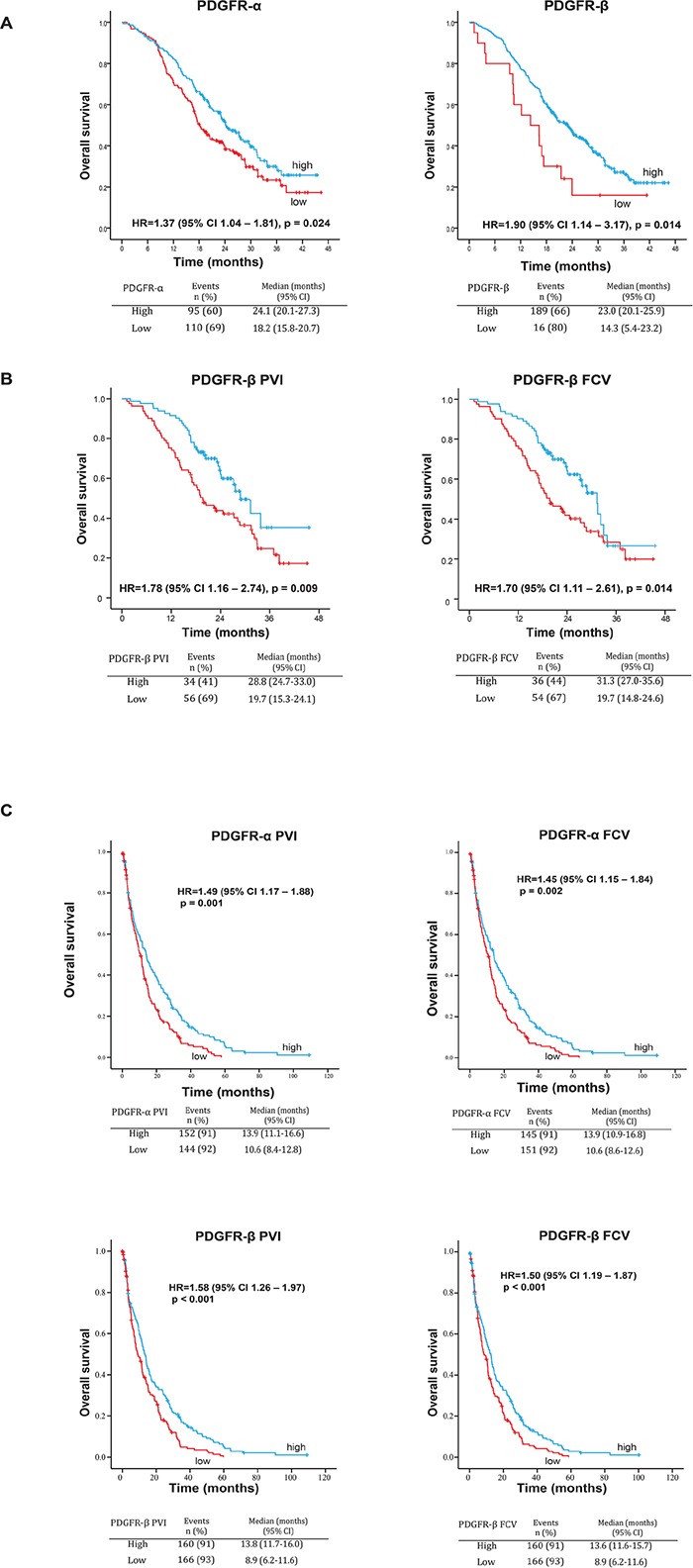
A. Associations between perivascular expression of PDGFR-α and PDGFR-β and OS in the NORDIC-VII cohort (manual scoring) Low expression of both PDGFR-α and PDGFR-β is associated with statistically significantly shorter OS in the total study population. **B. Associations between perivascular metrics PVI and FCV of PDGFR-β and OS or PFS in the NORDIC-VII cohort.** Low PVI and FCV of PDGFR-β are associated with statistically significantly shorter OS in the total study population. **C. Associations between perivascular metrics PVI and FCV of PDGFR-α and PDGFR-β and OS in the SPCRC cohort.** Low PVI and FCV of both PDGFR-α and PDGFR-β are associated with statistically significantly shorter OS in the total study population.

Low expression of either PDGFR-α or PDGFR-β was associated with significantly shorter overall survival (OS) (Figure 3A) with median OS 18.2 and 24.1 months for low and high PDGFR-α (p = 0.024) and 14.3 and 23.0 months for low and high PDGFR-β (p = 0.014), respectively. Low PDGFR-β expression was also associated with shorter progression free survival (PFS) (Supp. Item 6, upper panel, manual scoring).

These univariate analyses were expanded to a multivariate analysis, including performance status, alkaline phosphatase level and BRAF mutation status: three variables with marked prognostic importance [19–21]. Both perivascular markers acted as independent predictors of OS when tested individually in the analyses (Table 1).

**Table 1:** PDGFR-α and PDGFR-β as prognostic factors for overall survival in multivariate analyses in patients with mCRC (NORDIC-VII cohort; n=311)

	Adjusted with PDGFR-α		Adjusted with PDGFR-β	
Variable	HR (95% CI)	P value	HR (95% CI)	P value
**PDGFR-α (low *vs.* high)**	1.44 (1.09-1.91)	0.011	-	-
**PDGFR-β (low *vs.* high)**	-	-	1.82 (1.09-3.04)	0.023
**Alkaline Phosphatase (elevated *vs.* normal)**	2.00 (1.50-2.69)	<0.001	1.98 (1.47-2.65)	<0.001
**Performance status (1 *vs.* 0)**	1.92 (1.41-2.60)	<0.001	1.90 (1.40-2.57)	<0.001
**Performance status (2 *vs.* 0)**	4.02 (2.18-7.43)	<0.001	3.66 (1.99-6.73)	<0.001
**BRAF (mut *vs.* wt)**	2.94 (1.91-4.29)	<0.001	2.94 (1.09-3.04)	<0.001

Abbreviations: HR, hazard ratio; CI, confidence interval; mut, mutant; wt, wild type.

To qualify these findings the next tests were performed using PVI and FCV metrics derived from the digital image analyses of the double-staining, which included PDGFR-β, α-SMA or desmin as perivascular markers and CD34 as endothelial cell marker (for details see Materials and Methods, Supp. Methods and Supp. Item 1). PDGFR-α/CD34 double staining and related digital analysis was not performed on the material of the Nordic-VII cohort due to shortage of the available material. Analyses of associations between perivascular features and clinicopathological characteristics did not identify any significant associations (Supp. Item 7).

Notably, low PDGFR-β PVI and FCV were associated with significantly shorter OS. Median OS was 19.7 and 28.8 months for low and high PDGFR-β PVI (p=0.009) and 19.7 and 31.3 months for low and high PDGFR-β FCV (p=0.014), respectively (Figure 3B). Low PDGFR-β PVI and FCV were also associated with significantly shorter PFS, with median PFS 8.2 and 10.9 months for low and high PDGFR-β PVI, and 8,5 and 10,1 months for low and high PDGFR-β FCV (Supp. Item 6, upper panel, digital scoring). Neither α-SMA nor desmin showed statistically significant associations with OS or PFS (data not shown). Low vessel density was statistically significantly associated with shorter survival (Supp. Item 8).

Notably, both PDGFR-β-metrics acted as independent predictors of OS when tested individually in the multivariate analyses, which also included vessel density (Table 2).

**Table 2:** PVI and FCV of PDGFR-β as prognostic factors for overall survival in multivariate analyses in patients with mCRC (NORDIC-VII cohort, n=158)

	PVI		FCV	
variable	HR (95% CI)	P value	HR (95% CI)	P value
**PDGFR-β (low *vs.* high)**	1.78 (1.14-2.78)	0.011	1.79 (1.16-2.76)	0.009
**Vessel density (low *vs.* high)**	1.80 (1.16-2.78)	0.009	1.90 (1.23-2.90)	0.004
**Alkaline Phosphatase (elevated *vs.* normal)**	1.85 (1.20-2.84)	0.005	1.89 (1.23-2.90)	0.004
**Performance status (1, 2 *vs.* 0)**	1.67 (1.07-2.61)	0.023	1.65 (1.06-2.56)	0.027
**BRAF (mut *vs.* wt)**	4.48 (2.21-10.00)	<0.001	4.50 (2.23-9.07)	<0.001

Abbreviations: HR, hazard ratio; CI, confidence interval; mut, mutant; wt, wild type.

Together these analyses thus provide novel evidence for an association between perivascular PDGFR-β status and survival in mCRC.

### Perivascular status predicts survival in the SPCRC cohort of mCRC

Additional analyses were performed to investigate if the findings from the selected study population of NORDIC-VII could be reproduced in an independent un-selected population of mCRC patients. For this purpose digital-image-analyses-derived data on perivascular characteristics in the SPCRC cohort were analyzed with regards to clinicopathological characteristics and survival.

No significant associations were detected between perivascular PDGFR-α and PDGFR-β marker status and clinical characteristics such as WHO status, number of metastatic sites, alkaline phosphatase levels, gender or tumor location (Supp. Item 9 A and B).

In agreement with the findings from the NORDIC-VII cohort, statistically significant associations between low marker expression and short survival were found for all four PDGFR-related metrics (Figure 3C). Median OS for low and high PDGFR-α was 10.6 and 13.9 months (for both PVI and FCV with p=0.001 and 0.002, respectively). Median OS for low and high PDGFR-β PVI was 8.9 and 13.8 months (p<0.001). Similar results were observed in PDGFR-β FCV analyses (Figure 3C). Low PDGFR-α PVI and FCV, were also associated with significantly shorter PFS, with median PFS 7.1 and 8.9 months for low and high PDGFR-α respectively (Supp. Item 6, lower panel).

The perivascular markers also acted as statistically significant independent prognostic markers, as determined by multivariate analyses, with HRs ranging from 1.30 (PDGFR-α FCV) to 1.51 (PDGFR-β PVI) (Table 3).

**Table 3:** PVI and FCV of PDGFR-α and PDGFR-β as prognostic factors for overall survival in multivariate analyses in patients with mCRC (SPCRC cohort)

	Adjusted with PDGFR-α n=278				Adjusted with PDGFR-β n=307			
	PVI		FCV		PVI		FCV	
variable	HR (95% CI)	P value	HR (95% CI)	P value	HR (95% CI)	P value	HR (95% CI)	P value
**PDGFR-α (low *vs.* high)**	1.31 (0.01-1.70)	0.045	1.30 (1.00-1.68)	0.043	-	-	-	-
**PDGFR-β (low *vs.* high)**	-	-	-	-	1.51 (1.18-1.94)	0.001	1.44 (1.12-1.84)	0.004
**Alkaline Phosphatase (elevated *vs.* normal)**	1.63 (1.26-2.10)	0.001	1.63 (1.26-2.11)	<0.001	1.62 (1.26-2.08)	<0.001	1.61 (1.25-2.06)	<0.001
**Performance status (1, 2 *vs.* 0)**	1.45 (1.10-1.91)	0.008	1.48 (1.12-1.94)	0.005	1.47 (1.13-1.92)	0.004	1.48 (1.14-1.92)	0.004
**Performance status (3, 4 *vs.* 0)**	6.78 (4.44-10.35)	<0.001	6.76 (4.43-10.33)	<0.001	7.44 (5.01-11.03)	<0.001	7.43 (5.01-11.03)	<0.001
**BRAF (mut *vs.* wt)**	2.08 (1.46-2.94)	<0.001	2.13 (1.51-3.01)	<0.001	2.19 (1.59-3.01)	<0.001	2.21 (1.61-3.05)	<0.001

Abbreviations: HR, hazard ratio; CI, confidence interval; mut, mutant; wt, wild type.

The analyses of the SPCRC cohort thus established that the four perivascular metrics acted as independent markers of survival in a population-derived unselected cohort of mCRC.

### Perivascular PDGFR-β is concordant in primary tumor and in metastasis

Based on the nature of the NORDIC VII and the SPCRC cohorts, the analyses above focused on survival of metastatic CRC following earlier surgical removal of the primary tumor. Notably, marker status was collected from primary tumor tissue raising some concerns about biological significance of these findings. To address this issue, status of four vascular markers was compared in cases where information was available both from primary tumor and patient-matched metastasis.

**Figure 4:**
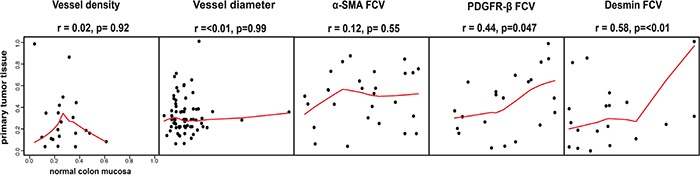
Associations of vessel size, vessel density or perivascular status between primary tumor and distant metastases Perivascular PDGFR-β and desmin status in primary tumor tissue and in metastatic tissue showed significant correlations. Notably, vessel density, vessel size and perivascular α-SMA did not show this correlation. Horizontal axes show tumor tissue data, vertical axes show metastatic tissue data. Spearman rank test used for statistical analysis.

As shown in Figure 4 markers showed a large variation in their degree of concordance. Notably only perivascular PDGFR-β and desmin showed a statistically significant concordance. Features such as vessel diameter, vessel density and perivascular α-SMA were not significantly correlated in this comparison of marker status in primary tumor and metastases.

Together these analyses suggest that the associations between perivascular PDGFR-β and survival, detected by analyses of primary tumor, is reflecting biological mechanisms likely to also be involved in the progression of metastatic disease.

## DISCUSSION

This study identified previously un-recognized inter-case heterogeneity of marker-defined perivascular cell subsets in CRC (Figure 1). According to our observation vessel density and perivascular PDGFR-α and -β are significantly associated with BRAF mutations status in the SPCRC cohort (Figure 2C). These findings should be further experimentally analyzed. Notably, BRAF-mutations have previously been reported to be associated with increased VEGF-A production [22]. Our study also identified strong associations between the perivascular PDGFR-β status of the tumor-vasculature and the host-vasculature in histologically normal tissue (Figure 2B). This suggests that genetic or life-style-related host-characteristics also contribute to inter-case perivascular heterogeneity. Similar findings have been made in prostate cancer regarding stromal PDGFR-β expression [23].

Notably, marker-defined perivascular subsets were independently expressed, potentially compatible with different regulatory mechanisms and functional differences between perivascular subsets, which should be further explored (Figure 1A-1B). Future analyses of clinical series should also consider other pericyte markers such as RGS5 and NG2. Furthermore, the analyses also showed that perivascular features are largely independent of vessel density and size (Figure 2A, Supp Item 5). This implies that perivascular status is governed by other factors than those determining vessel density and size, which should also be further analyzed in mechanistic studies.

Recent analyses of large datasets are emphasizing the contribution of stroma-derived genes to the definition of gene-expression-based molecular subsets of CRC [24, 25]. Future studies on tumor collections for which gene-expression data is available will allow further analyses on how the perivascular-defined groups of the present study are distributed among the gene-expression-based molecular CRC subsets.

A major finding of the present study is the associations between survival in mCRC and perivascular PDGFR status (Figure 3 and Tables 1–3). This finding is derived from analyses of two different mCRC cohorts, one including homogeneously treated trial patients and one unselected patients, with two independent IHC scoring methods. These associations remained statistically significant in multivariate analyses including three of the most important prognostic parameters in mCRC: performance status, alkaline phosphatase level and BRAF mutation status [19–21] (Tables 1–3).

All cases of NORDIC-VII and the majority of cases in the SPCRC received chemotherapy treatment. The study therefore does not allow a distinction between effects of perivascular markers on natural course of disease or response to treatment. Exploratory analyses of relationship of markers to PFS yielded some significant associations detected with PDGFR-α and PDGFR-β in the SPCRC and NORDIC-VII cohorts, respectively (Supp. Item. 6). No evidence for impact of perivascular status on cetuximab benefit was detected in analyses of the Nordic VII cohort (data not shown). Future studies are therefore warranted in other tumor collections in order to separately define the effects of perivascular cells on the natural course of the disease and on response to therapy.

Associations between pericyte status and survival have been reported in other tumor types. Analyses of patients with primary breast cancer noted, as in this study, an association between low pericyte coverage, determined by NG2 IHC, and poor prognosis [10]. In contrast, the analyses of serous ovarian cancer and renal cell cancer patients identified a statistically significant relationship between high pericyte coverage (determined by PDGFR-β and α-SMA respectively) and poor prognosis [17, 26].

The present study, restricted to analyses of clinical samples, does not address the mechanistic basis underlying the associations between survival and perivascular status. In the case of breast cancer, experimental studies have linked the reduced pericyte coverage with a hypoxic tumor state promoting an invasive HGF/c-met-dependent phenotype [10]. A more recent experimental breast cancer study linked low pericyte coverage to increased pro-metastatic Angiopoietin-2 signaling [16]. Other studies, using genetically modified mice, have suggested that reduced pericyte coverage is associated with an increased vascular permissiveness for intravasation [27]. Studies in mouse models have also suggested that the perivascular status will affect tumor immune surveillance [15]. Further correlative analyses should investigate to what extent these different mechanisms, identified in animal models, can explain the clinical associations of the present study.

Pre-clinical studies have indicated that pericyte-status affects survival signaling in endothelial cells and sensitivity to anti-angiogenic drugs, including VEGF-targeting agents [28–31]. Interestingly, correlative analyses of a neo-adjuvant breast cancer study just recently suggested that high perivascular coverage, as determined by perivascular ASMA quantification, was associated with benefit of bevacizumab [32]. It thus appears highly motivated to use, in future studies, the methods of the present study also to analyze impact of vascular and perivascular features on response of CRC to bevacizumab.

In conclusion, this study identifies novel independent markers associated with survival of metastatic CRC, highlights the relevance of perivascular cells in CRC tumor biology and thereby also suggests novel points for therapeutic interference.

## MATERIALS AND METHODS

A synopsis of the materials and methods is presented here. Full details are provided in the Supplementary Data.

Tissue microarrays (TMAs) from the NORDIC-VII cohort of mCRC [18] from the open-label randomized investigator-initiated, multicenter phase III trial (for details see Supp. Methods) were used for initial perivascular marker analyses.

The initial analyses were performed on 318 cases from NORDIC-VII cohort using single staining to PDGFR-α and PDGFR-β. The manual scoring and semi-quantitative four-grade system was used to evaluate perivascular expression of the markers (see Supp. Methods).

### Digital image analyses

To improve characterization of perivascular cells, a set of novel integrated analytical procedures were developed. These include double staining with antibodies to endothelial cell markers and pericyte markers and subsequent computerized analysis of digital images which was used to extract two “metrics” related to the perivascular staining: *Perivascular Intensity* (PVI) and *Fraction of Covered Vessels* (FCV), (for details see Supp. Methods).

The extended digital-image-analyses were performed on a subset of the 163 cases from the NORDIC-VII cohort (for the inclusion criteria see Supp. Methods) using three double staining with perivascular markers (one of PDGFR-β, α-SMA or desmin) and the endothelial cell marker CD34.

The SPCRC cohort of mCRC [33, 34], which represents an unselected population of all non-resectable histologically confirmed mCRC, was used for validation of findings from the NORDIC-VII cohort (Supp. Methods). Double staining with either of the perivascular markers PDGFR-α (n=323) or PDGFR-β (n=355) and the endothelial cell marker CD34 were made and results quantified by digital-image-analyses.

### Statistical analyses

All statistical tests were carried out using SPSS V20 (SPSS Inc., Chicago, IL). p values <0.05 were considered statistically significant (see Supp. Methods for details).

## SUPPLEMENTARY MATERIAL FIGURES AND TABLES


