# Supplementary material for: Survival-associated heterogeneity of marker-defined perivascular cells in colorectal cancer
Source: Oncotarget. 2016 May 26;7(27):41948–58. doi: 10.18632/oncotarget.9632 (PMC5173107; doi:10.18632/oncotarget.9632)
Supplement: Supplementary file 1 [file oncotarget-07-41948-s001.pdf]

Survival-associated heterogeneity of marker-defined perivascular cells in colorectal cancer

SUPPLEMENTARY DATA

Patient population

The NORDIC-VII study was an open-label randomized investigator-initiated, multicenter phase III trial. 571 patients were enrolled from 32 Nordic centers from May 2005 to October 2007. The intention-to-treat population consisted of 566 adult patients (18-75 years) with histologically confirmed mCRC (adenocarcinoma), no prior chemotherapy for advanced or mCRC, non-resectable and measurable disease according to the Response Evaluation Criteria in Solid Tumors (RECIST version 1.0), and WHO performance status (PS) ≤ 2 who were randomly assigned in a 1:1:1 ratio to receive either standard Nordic FLOX (arm A), cetuximab and Nordic FLOX (arm B), or cetuximab combined with Nordic FLOX intermittently (arm C). Details on study design, eligibility criteria, treatment and clinical results have been published [1].

The SPCRC cohort represents an unselected population of all non-resectable histologically confirmed mCRC patients from three Scandinavian health care regions during 2003-2006 [2]. Clinical characteristics, blood tests, treatments, response to treatment, progression-free survival and overall survival were recorded. The study included finally 798 patients. First-line chemotherapy was given to 456 patients (57%), 77% of these received combination chemotherapy. Median survival (95%CI) for all 798 patients was 9 (7.8-10.2) months, 15 (13.4-16.6) months for chemotherapy-treated patients and 2 (1.5-2.5) months for patients given best supportive care only.

Tissue microarray (TMA)

Tissue microarrays (TMAs) were made from routine, pre-treatment formalin-fixed, paraffin-embedded (FFPE) tissue blocks of primary tumor. For this study, TMAs from 318 patients from the NORDIC-VII cohort and from 429 patients from the SPCRC cohort were available for immunohistochemistry (IHC) analyses. For digital analyses on NORDIC-VII tissue material a subset of 163 cases was used. The major exclusion criteria were: tissue detachment from the glass of >50% of sample area, fat or muscle tissue of >50% of sample area, focusing issues of the slide scanning microscope which resulted on blurred digital image of >50% of sample area.

IHC procedures

Single staining

4 µm thick TMA sections were de-paraffinized, rehydrated and rinsed in distilled H_2O. After antigen retrieval with boiling in pH 9.0 retrieval buffer, sections were incubated with anti- PDGFR-α or PDGFR-β rabbit monoclonal antibody (#3164 and #3169, Cell Signaling Technology, Danvers, MA) 2µg/ml at dilution 1:100. For PDGFR-α and PDGFR-β detection sections were first incubated with a biotinylated secondary goat anti-rabbit antibody E0432, 1:500 (Dako Inc, Glostrup, Denmark) and then with the ABC-HRP kit (PK-6100, Vectastain ABC-HRP, Vector Laboratories, Burlingame, CA). Specific staining was visualized with diaminobenzidine (DAB) staining kit (SK100, Vector Laboratories). The sections were counterstained with hematoxylin.

Double staining

To develop double staining for perivascular markers (PDGFR-α, PDGFR-β, α-SMA and desmin) with CD34, TMA sections were de-paraffinized, rehydrated and washed in distilled H_2O as described above. After antigen retrieval in decloaking chamber (Biocare Medical) at 110 degrees C for 5 minutes in pH 10.0 (PDGFR-α or PDGFR-β) or pH 6.0 (α-SMA or desmin) retrieval buffer, sections were incubated with blocking solution for 30 min and then overnight with antibodies against PDGFR-α (#3164 Cell Signaling Technology, Danvers, MA at dilution 1:100), PDGFR-β (#3169 Cell Signaling Technology, Danvers, MA at dilution 1:100), α-SMA (Clone 1A4; Dako, Inc., Denmark at dilution 1:300) or desmin (Rabbit Anti-Desmin, Sigma-Aldrich Product Number HPA018803 at dilution 1:500). Sections were then incubated with polymer system (ImmPRESS™-AP Polymer Anti-Rabbit IgG MP-5401 or ImmPRESS™-AP Polymer Anti-Mouse IgG, MP-5402, Vector Laboratories, Burlingame, CA) for one hour at room temperature, and developed with Vector® Blue AP Substrate Kit (SK-5300, Vector Laboratories, Burlingame, CA). To destroy alkaline phosphatases reagents, sections were subsequently heated in decloaking chamber at 95 degrees C for 5 minutes, in pH 9.0 solution. After that

sections were incubated with blocking solution for 30 min and then overnight with anti-CD34 (Clone JC70A; Dako, Inc., Denmark) at dilution 1:100. Sections were then incubated with polymer system (ImmPRESS™-AP Polymer Anti-Mouse IgG, MP-5402, Vector Laboratories, Burlingame, CA) for one hour at room temperature, and developed with Vector® Red AP Substrate Kit (SK-5100, Vector Laboratories, Burlingame, CA). For the double staining with α-SMA and CD34 horse radix peroxidase-based amplification system (EnVision + System-HRP Dako A/S, EnVisionTM, Dako, CA, USA) were used for α-SMA and DAB for CD34 staining.

Triple staining

To develop triple staining for perivascular markers PDGFR-β, α-SMA and endothelial marker CD34, tumor sections were de-paraffinized, rehydrated and washed in distilled H_2O as described above. After antigen retrieval in decloaking chamber (Biocare Medical) at 110 degrees C for 5 minutes in pH 10.0 retrieval buffer, sections were incubated with blocking solution for 30 min and then overnight with antibodies against PDGFR-β (#3169 Cell Signaling Technology, Danvers, MA at dilution 1:100). Sections were then incubated with 0.5% blocking reagent (PerkinElmer Life Sciences, cat. no. FP1012) for 30 min and then with polymer system (EnVision + System-HRP Dako A/S, EnVisionTM, Dako, CA, USA) fro 30 min. After that sections were incubated with TSA Plus Cyanine 3.5system (PerkinElmer Life Sciences, cat. no. NEL763001KT) for 15 min. To destroy primary and secondary antibodies sections were heated in decloaking chamber at 90 degrees C for 5 minutes, in pH 9.0 solution.

The sections were incubated with blocking solution for 30 min and then overnight with antibodies against α-SMA (Clone 1A4; Dako, Inc., Denmark at dilution 1:300). Sections were then incubated with 0.5% blocking reagent (PerkinElmer Life Sciences, cat. no. FP1012) for 30 min and then with polymer system (EnVision + System-HRP Dako A/S, EnVisionTM, Dako, CA, USA) fro 30 min. After that sections were incubated with TSA Plus Cyanine 5 system (PerkinElmer Life Sciences, cat. no. NEL745001KT) for 15 min. To destroy primary and secondary antibodies sections were heated in decloaking chamber at 90 degrees C for 5 minutes, in pH 9.0 solution.

The sections were incubated with blocking solution for 30 min and then overnight with anti-CD34 (Clone JC70A; Dako, Inc., Denmark) at dilution 1:100. Sections were then incubated with 0.5% blocking reagent (PerkinElmer Life Sciences, cat. no. FP1012) for 30 min and then with polymer system (EnVision + System-HRP Dako A/S, EnVisionTM, Dako, CA, USA) fro 30 min. After that sections were incubated with TSA Plus Cyanine 5.5 (PerkinElmer Life Sciences, cat. no. NEL766001KT) for 15 min.

The sections was finally mounted with ProLong® Diamond Antifade Mountant with DAPI (Invitrogen).

Quantitative analysis of immunostaining and histochemical staining

Manual quantification of immunostaining

Single-antibody-stained slides (PDGFR-α or PDGFR-β) were scanned with the automated scanning system Aperio XT (Aperio Technologies, Inc.).Digital slides were viewed with the image viewer program ImageScope v. 11.1.2.752 (Aperio Technologies, Inc.). Digital images were evaluated with regard to staining intensity in perivascular area. The results were scored as negative (0), weak (1), moderate (2) or strong (3) (see Supplementary Item 2). For survival analyses these scores were used to dichotomize the cases in two groups.

Digital image analysis

The double-stained slides were scanned by a Vslide slide scanning microscope (Metasystems, Alltlussheim, Germany) using ×10 objective and RGB led illumination for color deconvolution.To view the scanned digital slides analyses the program Metaviewer (Metasysetms, Alltlussheim, Germany) was used. Tumor TMA sample images were manually annotated and saved in .tif format as individual images. The images were then proceeded to an automated image analysis platform.

An image-processing algorithm has been developed as a plugin for the ImageJ software (http://rsb.info.nih.gov/ij). The algorithm utilizes specific endothelial cell staining (for this study endothelial cells were marked with an antibody to CD34) to identify vessels. Perivascular areas are thereafter defined as a region which surrounds the vessel for a distance of 5.5 micrometers (see Supplementary Item 3A). After this region selection, the intensity of specific marker staining is measured in each perivascular area separately. Each case is thus linked with a list of vessels with values of perivascular marker intensity determined for each vessel.

Because of the RGB-based 8-bit format of the images, the intensity was measured in a scale of 256 grades (from 0 to 255). The intensity value is proportional to the amount of light transmitted through the slide. Since stained tissue absorbs the light the amount of the dye in the tissue is reciprocal to the intensity. The Beer–Lambert's law was used to calculate optical density (OD) of each area [3] which is proportional to the amount of light absorber.

Two related metrics were produced to characterise each cases. For the first metric, *Perivascular Intensity* (PVI), we calculated the average among the individual perivascular ODs for each case. For the second metric,

Fraction of Covered Vessels (FCV), vessels were dichotomized based on perivascular OD, for categorization of vessels into two groups: covered and uncovered. FCV was then determined as the ratio of covered vessels and total vessel in each case. Determination of PVI and FCV are further illustrated in Supplementary Item 3B and 3C).

Association of digital metrics with visual semi-quantitative scoring

To check if the developed automated image analyses method reveals perivascular characteristics, also detected by semi-quantitative manual scoring, analyses were done comparing the two types of scorings. For the validation of the digital quantification two independent observers performed semi-quantitative four-grade scoring of vessel density, vessel diameter and perivascular PDGFR-β status. Kruskal-Wallis test revealed significant concordance between visual and digital scores (Supplementary Item. 4).

Statistical analyses

For the NORDIC-VII cohort, OS was determined as the time from randomization of treatment of metastatic disease to time of death from all cases. OS was updated April 30, 2009 (1.5 year after included the last patient), and patients who were alive on this date were censored. PFS was defined as the time from randomization to the first documented disease progression, or death due to any cause, whichever occurred first.

For the SPCRC cohort OS was determined as the time from the diagnosis of distant metastasis to time of death irrespective of cause. OS was updated February 07, 2014 and patients who were alive on this date were censored.

The Kaplan-Meier method was used to compare OS and PFS in different groups. A Cox proportional hazards model was used to estimate relative hazards in both univariate and multivariate models. Kruskal-Wallis test was used for the comparative analyses of digital vs. manual scoring. For analyses of associations between marker expression and clinical characteristics, Mann–Whitney U test and ANOVA tests were used. All statistical tests were two-sided and P values <0.05 considered statistically significant. All statistical analyses were carried out using SPSS V20 (SPSS Inc., Chicago, IL).

REFERENCES

1. Tveit KM, Guren T, Glimelius B, Pfeiffer P, Sorbye H, Pyrhonen S, Sigurdsson F, Kure E, Ikdahl T, Skovlund E, Fokstuen T, Hansen F, Hofsli E, Birkemeyer E, Johnsson A, Starkhammar H, et al. Phase III Trial of Cetuximab With Continuous or Intermittent Fluorouracil, Leucovorin, and Oxaliplatin (Nordic FLOX) Versus FLOX Alone in First-Line Treatment of Metastatic Colorectal Cancer: The NORDIC-VII Study. J Clin Oncol. 2012; 30:1755-1762.
2. Sorbye H, Dragomir A, Sundstrom M, Pfeiffer P, Thunberg U, Bergfors M, Aasebo K, Eide GE, Ponten F, Qvortrup C, Glimelius B. High BRAF Mutation Frequency and Marked Survival Differences in Subgroups According to KRAS/BRAF Mutation Status and Tumor Tissue Availability in a Prospective Population-Based Metastatic Colorectal Cancer Cohort. Plos One. 2015; 10:e0131046.
3. Oda M, Yamashita Y, Nishimura G, Tamura M. Quantitation of Absolute Concentration Change in Scattering Media by the Time-Resolved Microscopic Beer-Lambert Law. Adv Exp Med Biol. 1994; 345:861-870.

Supplementary item 1: The summary of the staining and analytical methods performed on tissue cohorts, used for the study

cohort	PDGFR-α	PDGFR-β	α-SMA	desmin
**Nordic-VII**	S	S, D	D	D
**SPCRC**	D	D	-	-

S - single staining and semi-quantitative scoring.
D- digital staining and digital scoring.

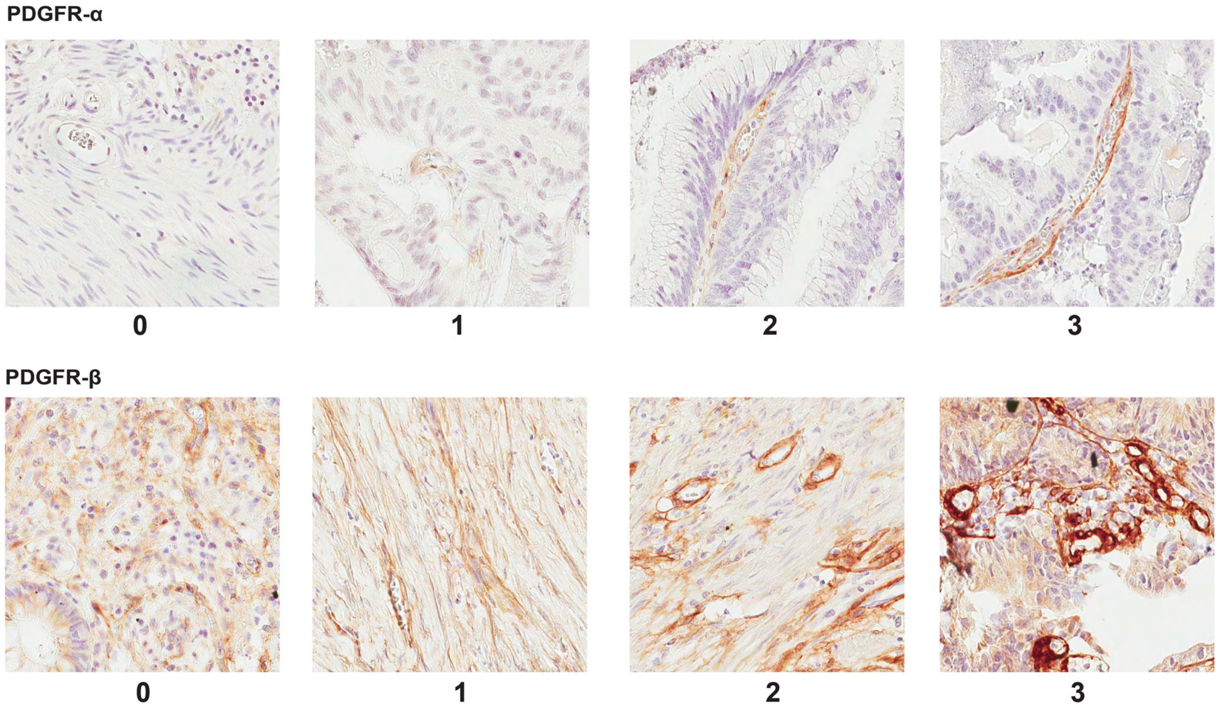

Supplementary item 2: Inter-tumor variability in perivascular expression of PDGFR-α and PDGFR-β. Representative images are shown to illustrate the four-grade scoring system. For each of the two perivascular markers DAB (brown) was used as a dye.

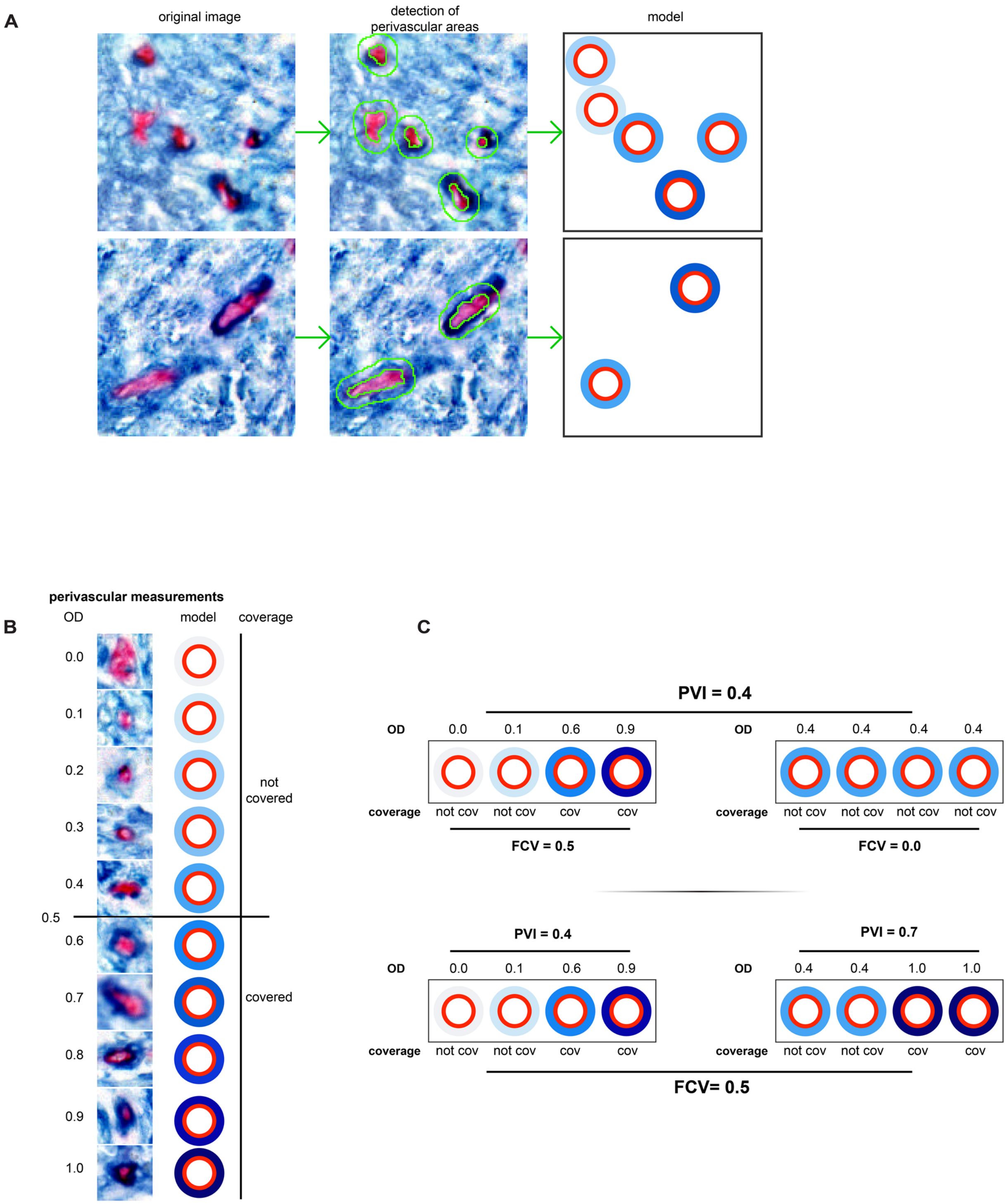

Supplementary item 3: Digital image analysis. A. Perivascular areas and marker expression in these areas are defined by the image analyses software. **B.** Original images and schematic illustration of tumors with different perivascular characteristics. Perivascular intensities of individual vessels are scored, as schematically illustrated in the left part with the 0.0-1.0 OD score. Individual vessels are also classified as 'uncovered' (OD below 0.5) or 'covered' (OD above 0.5) (right part of illustration). **C.** OD and coverage data are used to calculate perivascular intensity (PVI) and fraction covered vessels FCV. Upper part schematically illustrates two tumors with similar PVI, but different FCV, whereas lower part show two tumors with similar FCV but different PVI.

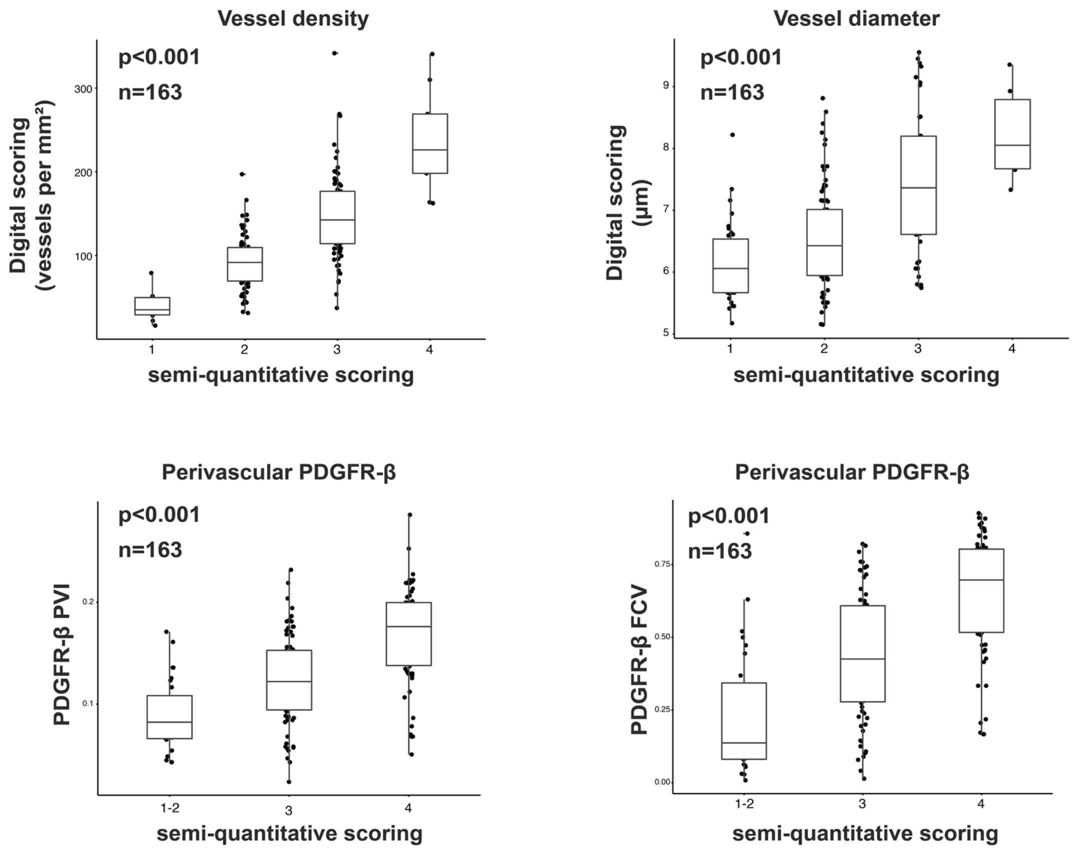

Supplementary item 4: Automated IHC measurements (Vessel density, Vessel Diameter, PVI and FCV) are concordant with visual semi-quantitative scores. Upper part: Box plots showing Vessel Density (upper left part) and Vessel Diameter (upper right part) digital measurements for tumors also classified in four classes by semi-quantitative scoring. Lower part: Box plots showing PVI (lower left part) and FCV (lower right part) for tumors classified as 0-1, 2 or 3 by semi-quantitative scoring for perivascular PDGFR-β status. Median values are shown by a *horizontal line*; the *box* represents values between the 25th and the 75th percentiles; and the *lower* and *upper bars* indicate non-outlier range. Statistical significance was assessed using Kruskal-Wallis test.

Supplementary item 5: Association between PVI of PDGFR-β, α-SMA or desmin with vessel diameter and distance to nearest neighbor vessel in primary colorectal tumors of NORDIC-VII

	Vdm		DNNV	
	**r**	**p**	**r**	**p**
α-SMA PVI		NS		NS
PDGFR-β PVI	0.0140	<0.001		NS
Desmin PVI	-0.0184	<0.001	0.0238	<0.001

Abbreviations: Vdm, vessel diameter; DNNV, distance to nearest neighbor vessel; r, Spearman's correlation coefficient; p, p value for Spearman nonparametric correlation; NS, not significant.

NORDIC-VII

manual scoring

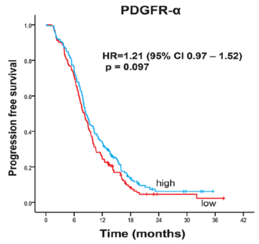

PDGFR-β PVI	Events n (%)	Median (months) (95% CI)
High	139 (88)	8.5 (7.6-9.1)
Low	148 (93)	8.1 (7.1-9.1)

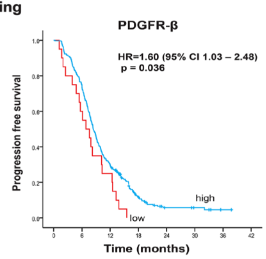

PDGFR-β PVI	Events n (%)	Median (months) (95% CI)
High	267 (90)	8.4 (7.7-9.0)
Low	20 (100)	6.8 (3.6-10.1)

digital scoring

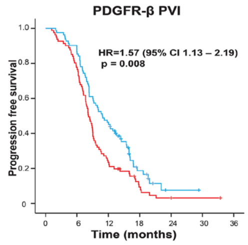

PDGFR-β PVI	Events n (%)	Median (months) (95% CI)
High	65 (79)	10.9 (8.4-13.3)
Low	76 (94)	8.2 (7.2-9.2)

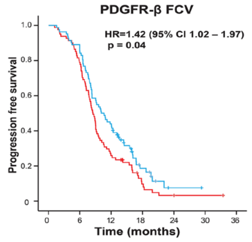

PDGFR-β FCV	Events n (%)	Median (months) (95% CI)
High	66 (81)	10.1 (7.7-12.5)
Low	75 (93)	8.5 (7.6-9.4)

SPCRC

digital scoring

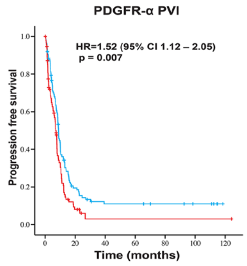

PDGFR-β PVI	Events n (%)	Median (months) (95% CI)
High	94 (83)	8.9 (8.0-9.9)
Low	81 (85)	7.1 (6.0-8.1)

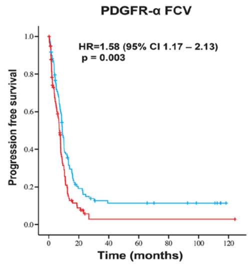

PDGFR-β PVI	Events n (%)	Median (months) (95% CI)
High	90 (83)	9.0 (7.7-10.3)
Low	85 (86)	7.1 (6.0-8.1)

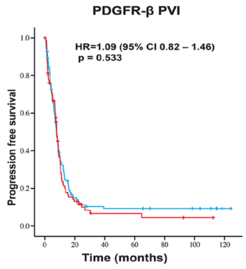

PDGFR-β PVI	Events n (%)	Median (months) (95% CI)
High	106 (86)	7.9 (6.5-9.3)
Low	83 (85)	8.0 (7.0-9.0)

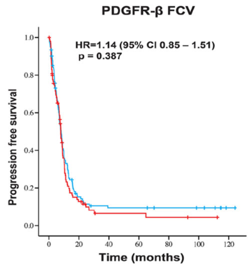

PDGFR-β PVI	Events n (%)	Median (months) (95% CI)
High	104 (85)	8.1 (6.6-9.5)
Low	85 (85)	8.0 (7.0-8.9)

Supplementary item 6: Associations between perivascular expression of PDGFR-α and PDGFR-β and PFS. Low perivascular expression of PDGFR-β analyzed by both semi-quantitative manual and digital scoring methods is associated with statistically significantly shorter PFS in NORDIC-VII cohort. Low perivascular expression of PDGFR-α but not PDGFR-β analyzed by digital scoring methods is associated with statistically significantly shorter PFS in SPCRC cohort.

Supplementary item 7: Association between PVI and FCV of PDGFR-β in primary colorectal tumors of NORDIC-VII with clinicopathological parameters

	PDGFR-β						
	PVI				FCV		
	n	mean	Std. dev	P value	mean	Std. dev	P value
WHO PS							
0	113	0.3266	0.1138		0.3642	0.2833	
1	45	0.3105	0.1175	0.622	0.3191	0.2699	0.554
2	5	0.3503	0.0520		0.4241	0.1454	
No. of metastatic sites							
1	51	0.3074	0.1192	0.208	0.3234	0.2857	0.288
>1	112	0.3300	0.1105		0.3673	0.2722	
Alk phosph							
normal	94	0.3177	0.1070	0.672	0.3456	0.2727	0.691
elevated	69	0.3300	0.1219		0.3644	0.2829	
Gender							
M	99	0.3330	0.1078	0.118	0.3777	0.2684	0.088
F	64	0.3073	0.1208		0.3163	0.2864	
Location							
colon	90	0.3326	0.1209	0.284	0.3833	0.2911	0.179
rectum	73	0.3109	0.1029		0.3170	0.2541	

Abbreviations: n, number of cases; std.dev, standard deviation; WHO PS, WHO performance status, Alk phosph, alkaline phosphatase; M, male; F, female; mut, mutant; wt, wild type.

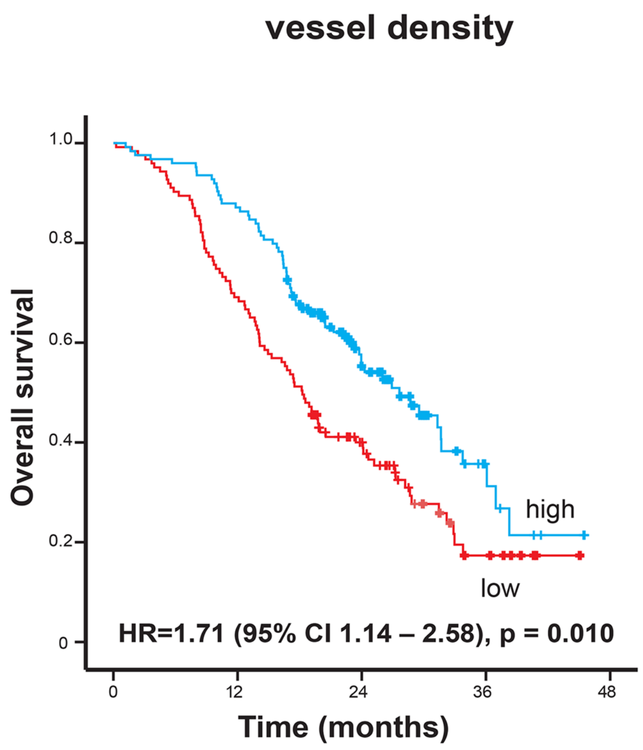

Supplementary item 8: Association between vessel density and OS in the NORDIC-VII cohort. Low vessel density is associated with statistically significantly shorter OS in the total study population.

Supplementary item 9: Association between PVI and FCV of PDGFR-α (A) and PDGFR-β (B) in primary colorectal tumors of SPCRC cohort with clinicopathological parameters

A

	PDGFR-α						
		PVI			FCV		
	**n**	**mean**	**Std. dev**	**P value**	**mean**	**Std. dev**	**P value**
WHO PS							
0	111	0.0150	0.0131		0.4556	0.3022	
1	108	0.0132	0.0103	0.144	0.3698	0.3269	0.059
2-4	119	0.0157	0.0123		0.4575	0.3327	
No. of metastatic sites							
1	145	0.0148	0.0119	0.919	0.4247	0.3226	0.829
>1	193	0.0146	0.0121		0.4321	0.3235	
Alk phosph							
normal	131	0.0144	0.0102	0.546	0.4471	0.3148	0.377
elevated	166	0.0149	0.0130		0.4185	0.3296	
Gender							
M	168	0.0144	0.0111	0.962	0.4299	0.3204	0.873
F	170	0.0149	0.0128		0.4278	0.3258	
Location							
colon	249	0.0142	0.0123	0.014	0.4064	0.3397	0.028
rectum	83	0.0159	0.0101		0.4966	0.3326	

B

	PDGFR-β						
		PVI			FCV		
	**n**	**mean**	**Std. dev**	**P value**	**mean**	**Std. dev**	**P value**
WHO PS							
0	124	0.0799	0.0439		0.4862	0.2747	
1	117	0.0747	0.0487	0.313	0.4348	0.2973	0.299
2-4	131	0.0780	0.0440		0.4818	0.2822	
No. of metastatic sites							
1	156	0.0780	0.0451	0.668	0.4803	0.2880	0.479
>1	216	0.0773	0.0457		0.4600	0.2827	
Alk phosph							
normal	143	0.0821	0.0429	0.057	0.5102	0.2716	0.046
elevated	184	0.0748	0.0455		0.4457	0.2870	
Gender							
M	182	0.0787	0.0428	0.317	0.4802	0.2751	0.389
F	190	0.0765	0.0479		0.4573	0.2940	
Location							
colon	275	0.0742	0.0413	0.073	0.4556	0.2778	0.140
rectum	91	0.0874	0.0544		0.5059	0.3032	

Abbreviations: n, number of cases; std.dev, standard deviation; WHO PS, WHO performance status, Alk phosph, alkaline phosphatase; M, male; F, female; mut, mutant; wt, wild type.
